# Environmental exposures associated with atopy in a rural community in Gwanda district, Zimbabwe: a cross-sectional study

**DOI:** 10.3389/fpubh.2024.1477486

**Published:** 2025-01-23

**Authors:** Vuyelwa Ndlovu, Moses Chimbari, Pisirai Ndarukwa, Elopy Sibanda

**Affiliations:** ^1^School of Nursing and Public Health, College of Health Sciences, Howard College Campus, University of KwaZulu-Natal, Durban, South Africa; ^2^Department of Environmental Science and Health, Faculty of Applied Sciences, National University of Science and Technology, Bulawayo, Zimbabwe; ^3^Department of Health Sciences and Faculty of Sciences and Engineering, Bindura University of Science Education, Bindura, Zimbabwe; ^4^Asthma, Allergy and Immune Dysfunction Clinic, Twin Palms Medical Centre, Harare, Zimbabwe; ^5^Department of Pathology, Medical School, National University of Science and Technology, Bulawayo, Zimbabwe

**Keywords:** allergic diseases, atopy, allergen sensitisation, environmental exposures, Zimbabwe

## Abstract

**Introduction:**

The increasing prevalence of allergic diseases in Zimbabwe may be attributed to changing environmental exposure patterns. In this study, we sought to identify the most influential environmental and lifestyle factors that may explain the observed atopy in a rural community in Zimbabwe.

**Methods:**

Using a cross-sectional study, information on a wide array of environmental and lifestyle exposures was self-reported by a sample of participants (children aged <18 years and adults aged ≥18 years) in the Gwanda district, Zimbabwe. To consenting participants, we performed skin prick testing (SPT) at a local clinic in Gwanda district to identify atopic individuals. Variables with a *p* value <0.25 from univariate analysis were included in backward-elimination multiple logistic regression analysis. Separate regression analyses were conducted for children (*n* = 108), adults (*n* = 388), and a subgroup of adults who reported ever being employed in any potentially harmful occupation (*n* = 153).

**Results:**

Compared with boys, girls were more likely to be sensitised to at least one allergen (OR = 4.87, 95% CI = 1.22–19.51). Among adults, the likelihood of sensitisation increased with increasing age (OR = 1.02, 95% CI = 1.01–1.03) and with a history of bloody urine and/or schistosomiasis (OR = 2.20, 95% CI = 0.98–4.95). In the subgroup of adults who reported ever being employed in any potentially harmful occupation, atopic sensitisation was associated with a history of tuberculosis (TB; OR = 3.37, 95% CI = 1.08–10.52) and a history of bloody urine and/or schistosomiasis (OR = 4.36, 95% CI = 1.40–13.65). Other notable, though not significant, factors were passive or parental smoking, alcohol consumption, indoor dampness and visible mould on walls.

**Conclusion:**

Girls were more likely to be sensitised to at least one allergen when compared to boys. Among adults, atopic sensitisation was positively associated with age, parental smoking, alcohol consumption and history of bloody urine or schistosomiasis but negatively associated with indoor cooking. A history of TB or helminth infection increased the likelihood of atopy among adults with history of employment. Longitudinal studies to explore the temporal and causal relationships between these factors and allergic outcomes are essential. There is a need for early public health interventions to address environmental and lifestyle factors for the prevention and control of allergic diseases in African rural communities.

## 1 Introduction

There is an increasing trend in the incidence of allergic diseases worldwide, and Zimbabwe has not been spared (1–3). The complex interplay of genetic, lifestyle and environmental factors has garnered considerable attention for explaining the increase in allergic diseases (4, 5). The genetic basis for susceptibility to allergic asthma and other allergic diseases is well established (6, 7). In recent years, several genes have been identified through genome-wide association studies (GWASs) in different population groups, including those of African ancestry (8, 9). Allergic sensitisation occurs when genetically susceptible (or atopic) individuals are exposed to a wide variety of environmental and lifestyle factors that are found in everyday life, such as household pests, air pollutants and the consumption of processed foods (10). Immunoglobulin E (IgE) antibodies that are produced upon exposure to environmental aeroallergens can be detected *in vivo* through a skin prick test (SPT) or *in vitro* via allergen-specific IgE and/or total serum IgE (11). Thus, IgE is considered an important biomarker for identifying atopy and allergic diseases triggered by environmental allergens (12).

We noted the case reports of *Imbrasia belina* allergy in Zimbabwe and Botswana with descriptions of symptoms serious enough to be life threatening (13, 14). *Imbrasia belina* or *Gonimbrasia belina*, is a popular indigenous edible insect, commonly referred to as ‘mopane worm’, and it taxonomically belongs to the *Saturniidae* family of the order *Lepidoptera* in the *Insecta* class. Mopane worm is widely distributed throughout Southern Africa and feeds mainly on the leaves of the mopane tree (*Colophospermum mopane*) (15–17). In Zimbabwe, mopane worm distribution is mostly concentrated in the southern parts of the country, particularly in Gwanda district. Rural communities in the area traditionally harvest mopane worm during the rainy season. The practice has, over time, increased to meet the growing commercial demand and it is not uncommon for the entire family to participate including children that are deemed old enough (18). This has raised concerns of increased potential for allergic diseases in the community (18). Although rural settings in Africa are considered to be at a lower risk of allergic diseases than urban communities are (19–21), the risk in the former is gradually increasing. We speculated at the possibility of mopane worm allergy being more than a few isolated case reports and designed the Gwanda Asthma and Respiratory Allergy Study (GARAS) set in Garanyemba, a rural community with a total population of 7,984 people in Gwanda district (18). The purpose of the study was to estimate the level of sensitisation and the clinical relevance of *Imbrasia belina* (mopane worm). Sensitivity to allergens was assessed using a panel of locally relevant aeroallergens that included 10 different commercial allergen extracts (Stallergenes, France) and in-house extracts of mopane worm *(Imbrasia belina)* and mopane tree leaves (*Colophospermum mopane*) (22). Skin prick tests were performed on eligible and consenting participants at a local clinic in Garanyemba especially selected for the study (18, 22). The prevalence of sensitisation, in order of highest to lowest, to each of the specific allergen extracts considered for this study were as follows: mopane worm (14.29%), *Tyrophagus putrescentiae* (14.29%), mopane tree leaves (13.42%), *Alternaria alternata* (6.49%) and *Dermatophagoides pteronyssinus* (6.49%), Barley grass (5.63%), Tree mixture (Maple, Horse chestnut, Plane, False acacia, Lime; 5.63%), Cockroach (5.41%), *Dermatophagoides farinae* (4.98%), Mosquito (4.76%), Weed mixture (4.11%) and Five Grass mixture (cocksfoot, meadow, rye, sweet vernal, timothy; 2.81%). The majority of the participants, particularly women, were polysensitised. Upon examining the sensitisation patterns, we found that the allergens that tended to cluster together in adults were mopane worm, mopane leaves and *Tyrophagus putrescentiae*. Co-sensitivity, among children, was frequently between mopane worm and mopane tree leaves. While there was frequent co-sensitisation between mopane worms and mopane leaves, possibly due to co-exposure, there were cases of monosensitivity to both extracts. The frequent co-sensitisation to mopane worm and *Tyrophagus putrescentiae* extracts could have been due to cross-reactivity to pan-allergens such as tropomyosin and arginine kinase (23). Co-exposure between the allergens was also a possible explanation as *Tyrophagus putrescentiae* (24) may have contaminated mopane worm during storage (25). Data were collected on harvesting practices and using adjusted regression analyses, we observed that mopane worm sensitisation was associated with mopane worm exposure mainly through occupational means (22). A manuscript summarising our findings on the clinical relevance of mopane worm sensitisation is underway.

While exposure to common allergens is an important risk factor for allergic diseases (6, 26), it is insufficient to explain the surge in allergen sensitisation and, subsequently, that of allergic diseases. It is believed that allergen-specific sensitisation may be influenced not only by exposure to the allergen sources in question but also by other background environmental exposures that may modify the impact of allergens. Studies have shown that other environmental contaminants, such as microbes, air and chemical pollutants, act as adjuvants in several stages of the allergen sensitisation pathway (27). Additionally, environmental and lifestyle factors, including prenatal maternal smoking, inhalant pollutants, psychosocial stress, diet and a lack of physical exercise, have been found to induce epigenetic changes in susceptibility genes for allergies (28, 29). Increased exposure to these factors is due to environmental and lifestyle changes that are best described and summarised by the emerging Anthropocene concept, an approach to understanding the influence of human activities on Earth’s natural systems (30). At present, relatively few studies in Africa have investigated allergic diseases, and much of what is known about their inception and phenotypic expression is derived from developed countries (31–33). Peculiarities in the natural history of allergies in African populations are thus expected due to the underlying differences in genetic, environmental and lifestyle characteristics compared to those of other continents (31, 34–36).

The sensitisation patterns from the GARAS study indicated high levels of polysensitisation coupled with age and gender differentials that we believe were suggestive of the influence of other environmental factors (22). These exposures need to be identified and taken into consideration when assessing the clinical relevance of mopane worm sensitisation. Thus, in the present study, we sought to identify the most important risk factors for atopy from a wide array of self-reported environmental exposures in childhood and adulthood among villagers in Garanyemba, Gwanda district.

## 2 Materials and methods

### 2.1 Study design and setting

The GARAS recruitment and data collection protocol has previously been described (18). This cross-sectional study was conducted in Gwanda District between October 2019 and March 2020. The Gwanda District is a semiarid area whose main economic activities include mining, livestock ranching and cultivation of drought-tolerant small grains such as sorghum (37). Malnutrition and infectious diseases such as malaria, schistosomiasis and tuberculosis (TB) are common problems even though efforts have been made to control them (38). The district has substantial mineral reserves, which make mining (both large and small scale) a major economic activity (39). An increase in artisanal and small-scale miners has had a significant negative effect on the environment, and there have been reports of health effects among residents in mining areas (39, 40).

Children aged 10–17 years and adults aged 18 years and above were recruited from each of the 8 villages in Garanyemba, Gwanda district. To be eligible, they had to have been residing in the study area for at least a year. A sample size of 496 participants was calculated on the basis of a mopane worm sensitization estimate of 50%, a 5% margin of error at 95% confidence interval and an additional 20% to compensate for anticipated non-response. Nonprobability sampling (volunteer sampling) was achieved with the assistance of the village leaders from each of the eight villages. The decision to utilise volunteer sampling was informed by the low response rate in the feasibility study when random sampling was employed (18). Written informed consent was obtained from adults or their legally authorised representatives (LAR) where appropriate, and in the case of children, parental consent and child assent were obtained. The study was approved by the Medical Research Council of Zimbabwe (Ref number MRCZ/A/2486) and the University of KwaZulu-Natal’s Biomedical Research Ethics Committee BREC (Ref number BE 327/19).

### 2.2 Sensitisation to inhalant allergens

Skin prick tests were performed by trained staff at the clinic using House Dust Mites (*Dermatophagoides farinae* and *Dermatophagoides pteronyssinus*), a storage mite (*Tyrophagus putrescentiae*), Tree mixture, Five Grass mix, Weed mixture, Barley grass, Cockroach, Mosquito, *Alternaria alternata,* mopane worm *(Imbrasia belina)* and mopane tree leaves (*Colophospermum mopane*). Histamine (10 mg/mL) and saline (0.9% NaCl) were included in the panel as positive and negative controls, respectively. Commercial allergen extracts (Stallergenes, France) were used with the exception of mopane worm and mopane leaf in-house extracts, which were prepared using previously described procedures (18). A wheal diameter exceeding 3 mm or greater than the saline control was considered positive for allergic sensitisation. A participant was classified as atopic if sensitised to at least one allergen (41).

### 2.3 Demographic and environmental factors

During field days, participants presented at the clinic that was selected for all data collection (18). Upon arrival, questionnaires were administered to consenting participants by trained interviewers in the local language (isiNdebele) using Kobo Collect software (42). The questionnaire included questions pertaining to socio-demographic and lifestyle characteristics (age, sex, education, occupation, monthly household income, smoking and alcohol consumption) and health-related characteristics (self-reported history of allergy, tuberculosis and schistosomiasis).

Data on a wide range of environmental exposures were collected across the following domains: (1) Early life environmental and lifestyle exposures, (2) Current exposures in and around the home and (3) Current exposures in the workplace. The exposure questionnaire gathered information on early life environmental experiences during childhood which included place of birth, family history, physical characteristics of the home, schooling, diet, passive smoking, infections, and outdoor exposures. Current indoor exposures such as parental or passive smoking, visible mould on walls, leaky roofs, indoor cooking with firewood and the presence of household pests such as rats and cockroaches in the past month were also collected. Questions regarding exposures in the workplace, directed at the adults, included employment status, type and duration of occupation, occupational exposure to dust or fumes and use of Personal Protective Equipment (PPE).

The majority of environmental exposure questions used in this study were extracted from the previously validated International Study of Asthma and Allergies in Childhood (ISAAC) and (43) and The European Community Respiratory Health Survey (ECRHS) (44) questionnaires. However, to fully capture environmental exposure experiences in Gwanda based on characteristics such as economic activity patterns, places of residence, eating habits and prevailing diseases, some relevant questions that were not available in the validated questionnaires were added to the GARAS questionnaire. We included questions on the history of TB (Have you ever suffered from TB?) and schistosomiasis (Have you ever suffered from schistosomiasis/ bloody urine?) in the questionnaire because of schistosomiasis endemicity and the high TB burden in Gwanda district (38, 45). The relationship between these infectious diseases and atopy has been explored in other studies (46, 47). Additionally, the burden of TB and of diseases linked to particulate matter exposure such as silicosis has been found to be particularly high among artisanal and small-scale miners in Zimbabwe (48). This led us to consider artisanal miners as a vulnerable population group potentially at higher risk of allergic diseases than other adults in the study population because of the difficult and harmful conditions they work in. For this reason, a question “Do you currently work in mining (Artisanal/Small scale/employed by a big mine)?,” was included in the occupational exposure section of the questionnaire. Questions addressing current dietary habits for both adults and children and recall of childhood dietary habits for adults were also included in the questionnaire. Prior to asking the question, interviewers briefly defined and explained the difference between traditional/indigenous foods and processed/modern foods. Traditional or indigenous foods were defined as flora and fauna that have been part of the local food system for generations and have social and cultural value (49). A few local examples that were given include *Adansonia digitata L.* (baobab fruit), *Berchemia discolour* (bird plum fruit), *Pennisetum typhoides* (pearl millet), *Voandzeia subterranea* (groundnuts), dried groundnuts, *Vigna unguiculata* (cowpeas and the leaves) and *Imbrasia (Gonimbrasia) belina* (mopane worms) (50). Adults were asked to recall their childhood dietary habits and answer the question “How would you best describe the diet during your childhood?” A follow-up question to describe current dietary habits, “How would you best describe your diet in the last 12 months?” was answered by both adults and children. Response options to both questions were: (1) mostly traditional, (2) combination of traditional and processed or fast foods and (3) mostly processed or fast foods.

### 2.4 Statistical analysis

Socio-demographic, environmental and health -related data were summarised and presented using descriptive statistics. Numerical variables were expressed as mean and standard deviation or median and interquartile range (IQR) depending on normality status using the Shapiro-Wilks test. Categorical variables were summarised as frequencies and percentages. Descriptive analyses were stratified by age (adults and children) and sex due to expected differences in environmental exposure patterns (51). The differences between adults and children were tested for statistical significance using the chi-square test or Fisher’s exact test for categorical variables; in the case of numerical variables, the two sample t test or Wilcoxon rank-sum test was used. Prior to multivariate analysis, the Variance Inflation Factor (VIF) was calculated to assess multicollinearity and none was detected.

Multivariate logistic regression was used to estimate the odds ratios (ORs) and 95% confidence intervals (CIs) to determine the associations between environmental and lifestyle characteristics and atopic sensitisation. We developed separate models for children, all adult participants and a subgroup of adults with a history of or current employment in a potentially harmful occupation. The occupational setting was deemed an important source of harmful environmental exposures in this study due to the nature of the main economic activities occurring in the Gwanda district (37). In each subgroup, unadjusted logistic regression analysis was initially performed to assess the relationship between each of the socio-demographic and environmental exposure variables and atopic sensitisation. Variables from the unadjusted analysis with *p* ≤ 0.25 and those of clinical significance were included in a multivariate model implementing a backward elimination process (52, 53). Age and sex were added *a priori* to all the models because of the differential environmental exposure patterns and susceptibility to allergic disease outcomes that have been observed (51, 54). Model fit was assessed using the Hosmer–Lemeshow test, with a *p* value >0.05 suggesting good overall fit (55). Analyses were performed using Stata version 13 (StataCorp, College Station, TX, United States), and *p* values less than 0.05 were considered to indicate statistical significance.

## 3 Results

**Figure 1:**
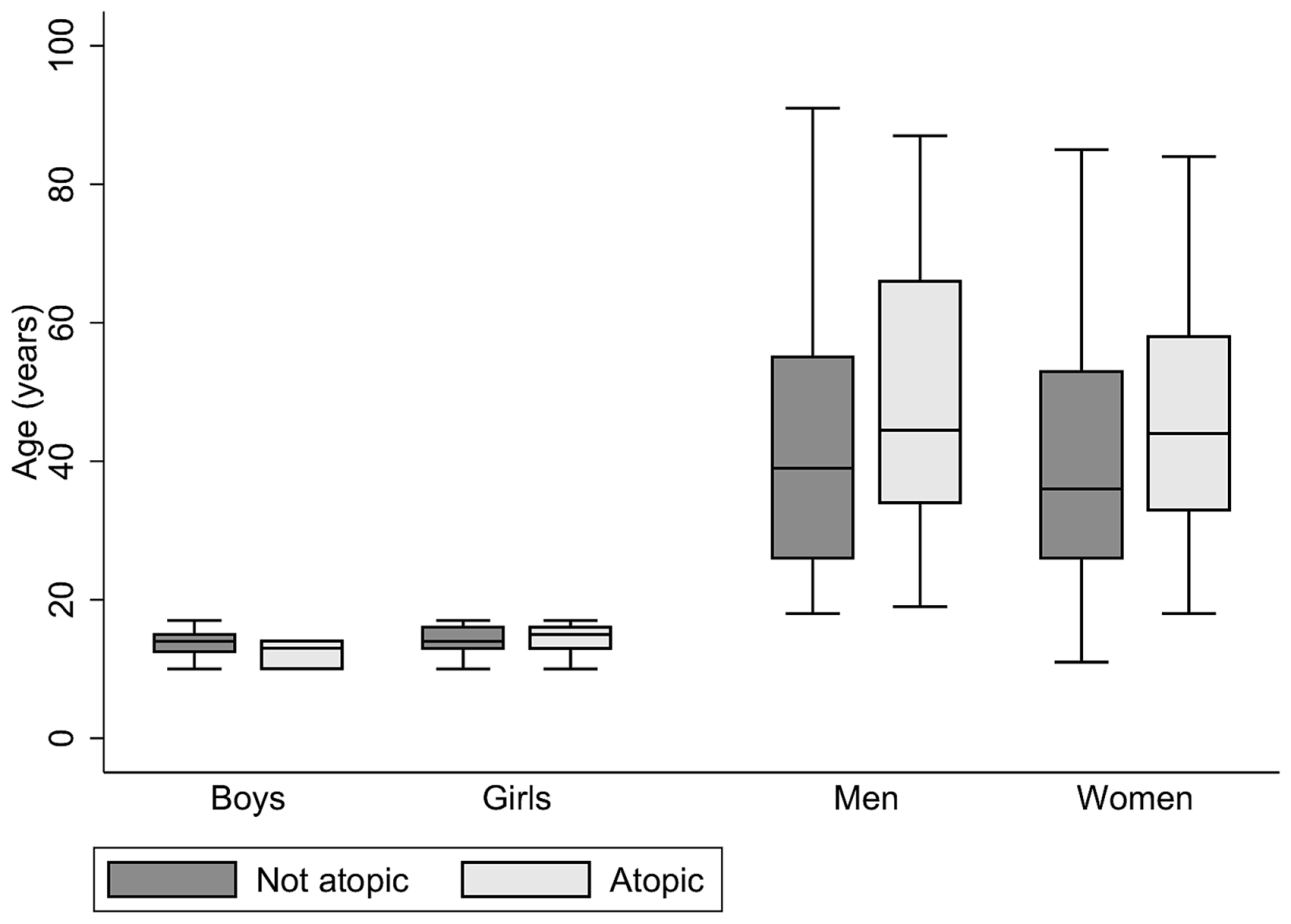
Age and sex distribution by atopic status in the study. Mean age (years) ± sd in the non-atopic group: boys, 13.82 ± 1.96; girls, 14.24 ± 1.87; men, 42.37 ± 18.76; women, 40.57 ± 18.06. Mean age (years) ± sd in the atopic group: boys, 12.33 ± 2.08; girls, 14.29 ± 2.35; men, 48.71 ± 20.03; women, 46.10 ± 17.04 (atopic women are significantly older than non-atopic women, *p* = 0.01).

Of the 496 individuals included in the GARAS study, 388 (78%) were adults (aged ≥18 years), and 108 (22%) were children (aged <18 years). A summary of the socio-demographic and health-related characteristics of the study participants is presented in Table 1. The mean age was 42.84 (±18.06) years for adults and 14.09 (± 2.03) years for children. There were more females than males among both adults and children. Self-reported ever smoking and alcohol consumption were reported by 13.35% and 15.79%, respectively, of the adults. Self-reported nasal allergies and skin allergies were more prevalent in adults than in children. The prevalence of a history of tuberculosis among adults was 8.90%. A response rate of 93.15% was achieved for skin prick testing, and the overall prevalence of atopic sensitisation was 31.17%. Furthermore, the prevalence of atopic sensitisation was greater in adults (33.33%) than in children (23.53%; *p* = 0.059). The distribution of atopic status by sex and age is illustrated in Figure 1. While there were no significant age differences by atopic status in children, atopic women were significantly older than non-atopic women (*p* = 0.01).

**Table 1:** Socio-demographic and health-related characteristics among adults and children in a rural community in Gwanda, Zimbabwe.

Characteristic	Adults (*n* = 388)	Children (*n* = 108)	Total
Socio-demographic characteristics			
Age, mean(sd)	42.84 (18.06)	14.09 (2.03)	36.58 (19.92)
**Sex, *n* (%)**			
Male	115 (29.64)	34 (31.48)	149 (30.04)
Female	273 (70.36)	74 (68.52)	347 (69.96)
**Level of Education** **(*n* = 490), *n* (%)**^a^			
No education	23 (6)	0 (0)	23 (4.69)
Primary	147 (38.48)	34 (31.48)	181 (36.94)
Secondary and tertiary	212 (55.50)	74 (68.52)	286 (58.37)
Monthly household income below poverty line (*n* = 354), n (%)^a^^,^^b^	354 (100)	–	–
Ever smoked	51 (13.35)	1 (0.95)	52 (10.68)
Alcohol use	60 (15.79)	0 (0)	60 (12.3)
Health related characteristics, n (%)			
Atopy (*n* = 462), n (%)^**c**^	120 (33.33)	24 (23.53)	144 (31.17)
History of allergy among parents, n (%)	85 (22.31)	13 (12.04)	98 (20.04)*
History of allergy among siblings, n (%)	124 (32.63)	20 (18.52)	144 (29.51)*
Nasal allergy, n (%)	107 (28.01)	23 (21.3)	130 (26.53)
Skin allergy, n (%)	59 (15.49)	11 (10.19)	70 (14.31)
History of Tuberculosis	34 (8.90)	1 (0.93)	35 (7.14)*
History of bloody urine/Schistosomiasis	32 (8.38)	2 (1.85)	34 (6.94)*

a There were missing data (n < 496) in some variables.

b The World Bank’s International Poverty Line of US$1.90/person/day was used. Children were not asked about monthly household income.

c 462 participants consented to SPTs and atopy was more common in adults than children (*p* = 0.0590).

*p < 0.05.

There were differences in the prevalence of environmental factors between adults and children (Table 2). Adults reported a greater median number of siblings (6, IQR = 4–8) compared to children (4, IQR = 3–6). Exposure to environmental tobacco smoke (ETS), reported as exposure to parental smoking or passive smoking, was more common in adults than in children. Adults were asked to recall and describe their childhood dietary habits, and 77 (20.16%) reported consuming a predominantly traditional diet. A follow-up question to describe dietary habits over the last 12 months revealed that none of the children and 9 (2.36%) adults were currently eating a predominantly traditional diet. The use of firewood for cooking was reported by the majority of participants, and most of the cooking was done indoors (83.67%). Among the adults who had ever been employed, 37 (24.34%) were artisanal miners.

**Table 2:** Summary of environmental exposures among children and adults.

Characteristic, *n* (%)	Adults (*n* = 388)	Children (*n* = 108)	Total
Born in rural areas	338 (88.95)	96 (88.89)	434 (88.93)
Number of siblings, median(IQR)*	6 (4–8)	4 (3–6)	6 (4–8)
Parental smoking*	126 (32.98)	21 (19.44)	147 (30)
Passive smoking	196 (51.31)	36 (33.96)	232 (47.54)
Housing characteristics			
Traditional housing	361 (94.5)	99 (91.67)	460 (93.88)
Brick housing	21 (5.50)	9 (8.33)	30 (6.12)
Leaking roof	73 (19.21)	19 (17.76)	92 (18.89)
Visible mould on walls*	86 (22.51)	12 (11.11)	98 (20)
Household pests*	195 (51.05)	41 (37.96)	236 (48.16)
Childhood diet			
Traditional	77 (20.16)		
Mixed	301 (78.80)		
Processed	4 (1.05)		
Diet in the last 12 months			
Traditional	9 (2.36)	0 (0)	9 (1.84)
Mixed	363 (95.28)	107 (99.07)	470 (96.11)
Processed	9 (2.36)	1 (0.93)	10 (2.04)
Cooking area			
Indoors	323 (84.55)	87 (80.56)	410 (83.67)
Outdoors	59 (15.45)	21 (19.44)	80 (16.33)
Heat source for cooking			
Firewood	378 (98.95)	108 (100)	486 (99.18)
Electricity	4 (1.05)	0 (0)	4 (0.82)
Employment history			
Ever employed (*n* = 382)	153 (40.5)	–	–
Artisanal mining (*n* = 153)	37 (24.2)	–	–

*p < 0.05.

Unadjusted logistic regression analysis was performed separately for adults and children to assess the relationship between each of the socio-demographic and environmental exposure variables and atopic sensitisation (Table 3). For children, the selected independent variables were age; sex; passive smoking; leaking roof; and the presence of household pests such as cockroaches and rats. All these variables were included in the multivariate analysis, and the results are presented in Table 4. Girls were significantly more likely to be sensitised to at least one allergen compared to boys (OR = 4.87, 95%CI = 1.22–19.51). Passive smoking, a leaking roof and presence of household pests also appeared to increase the risk of sensitisation among children, but these findings were not statistically significant.

**Table 3:** Unadjusted associations between environmental exposures and atopic sensitisation among children and adults.

	Children		Adults	
Variables	OR(95% CI)	*p* value	OR(95% CI)	*p* value
Socio-demographic characteristics				
Age	0.99 (0.79–1.24)	0.92	**1.02 (1.01–1.03)**	**0.005**
Sex	**3.92 (1.07–14.31)**	**0.04**	0.91 (0.56–1.46)	0.69
Siblings	0.91 (0.75–1.11)	0.36	1.02 (0.94–1.10)	0.61
Primary education	1.53 (0.58–4)	0.39	0/67 (0.29–1.53)	0.34
Secondary and tertiary education	–	–	0.63 (0.28–1.40)	0.26
Income	–	–	1.56 (0.64–3.84)	0.33
Ever smoker	–	–	**1.70 (0.92–3.16)**	**0.09**
Parental smoking	1.4 (0.47–4.13)	0.54	**1.58 (0.99–2.51)**	**0.055**
Passive smoking	**1.83 (0.72–4.68)**	**0.21**	1.04 (0.67–1.62)	0.85
Alcohol	–	–	**1.88 (1.06–3.35)**	**0.03**
Health related characteristics				
History of allergy among parents	0.97 (0.24–3.86)	0.97	**1.36 (0.80–2.31)**	**0.25**
History of allergy among siblings	1.2 (0.38–3.77)	0.75	**1.37 (0.86–2.2)**	**0.19**
History of Tuberculosis	–	–	**2.05 (1.01–4.22)**	**0.05**
History of bloody urine/Schistosomiasis	–	–	**2.53 (1.16–5.52)**	**0.019**
Exposures in and around the home				
Born in rural area	0.8 (0.19–3.29)	0.76	0.95 (0.45–1.98)	0.89
Livestock			1.19 (0.68–2.10)	0.55
Type of housing	0.52 (0.06–4.56)	0.56	1.55 (0.52–4.57)	0.43
Leaking roof	**2 (0.65–6.15)**	**0.23**	1.14 (0.65–2.01)	0.65
Visible mould on walls	0.62 (0.13–3.04)	0.55	**1.50 (0.89–2.52)**	**0.13**
Presence of household pests	**1.68 (0.67–4.25)**	**0.25**	**1.37 (0.88–2.14)**	**0.16**
Childhood diet	0.26 (0.02–3.61)	0.32	0.79 (0.46–1.34)	0.38
Diet in the last 12 months	–	–	0.62 (0.21–1.81)	0.38
Indoor cooking	1.07 (0.53–2.14)	0.85	**0.56 (0.31–1.01)**	**0.06**
Firewood for cooking	–	–	0.49 (0.07–3.52)	0.48
Exposures in the workplace				
Artisanal mining	–	–	**0.59 (0.26–1.38)**	**0.23**

Variables from unadjusted regression analysis with *p* ≤ 0.25 and those of clinical significance were deemed eligible for inclusion in multivariate analysis (results in boldface for selected variables). – Odds ratios were not determinable among children for a number of variables due to either perfect prediction of outcome or collinearity.

**Table 4:** Multivariate analysis of host-related and environmental risk factors for atopy among children.

Variables	OR(95% CI)	*p* value
Age	1.01 (0.79–1.29)	0.94
Gender	4.87 (1.22–19.51)	0.025
Passive smoking	1.93 (0.70–5.30)	0.20
Leaky roof	2.03 (0.57–7.24)	0.27
Presence of household pests	1.66 (0.59–4.65)	0.34

For adults, the selected independent variables in the unadjusted logistic regression analysis were age, sex, parental smoking status, alcohol consumption, history of bloody urine, visible mould on walls and indoor cooking (Table 3). The results of the adjusted logistic regression analysis with backward elimination showed that the likelihood of sensitisation increased with increasing age (OR = 1.02, 95%CI = 1.01–1.03). Adults reporting a history of bloody urine or schistosomiasis were two times more likely to be sensitised to at least one allergen compared to those who did not have such a history (*p* = 0.056). On the other hand, atopic sensitisation was less likely among those who cooked indoors compared to outdoor cooking (OR = 0.69, *p* = 0.07; Table 5).

**Table 5:** Multivariate analysis of host and environmental risk factors for atopy among all adults and the subgroup of adults who reported having occupations outside the home.

	All adults		Adults with occupations outside the home	
Variables	OR(95% CI)	*p* value	OR(95% CI)	*p* value
Age	1.02 (1.01–1.03)	0.015	1.01 (0.99–1.03)	0.35
Sex	1.47 (0.81–2.66)	0.20	1.38 (0.56–3.40)	0.49
Parental smoking	1.54 (0.95–2.50)	0.08	–	–
Alcohol	1.88 (0.93–3.78)	0.08	–	–
History of Tuberculosis	–	–	3.37 (1.08–10.52)	0.037
History of bloody urine/Schistosomiasis	2.20 (0.98–4.95)	0.056	4.36 (1.40–13.65)	0.01
Visible mould on walls	1.53 (0.88–2.67)	0.13	1.93 (0.75–4.94)	0.17
Indoor cooking	0.69 (0.47–1.03)	0.07	–	–
Artisanal mining	–	–	0.56 (0.21–1.46)	0.23

Among the adults who were previously employed, a wide range of occupations were reported, including mining, domestic work, farming, cross-border trading and construction. The most frequently reported occupation was artisanal mining 37 (24.34%). We considered all these to be potentially harmful according to reports from occupational health studies (56). After restricting the adult population to those that reported having had an occupation outside the home, logistic regression analysis was repeated using the same covariates selected for inclusion in the total adult population. The results, as presented in Table 6, showed that atopic sensitisation was associated with a history of TB (OR = 3.37, 95%CI = 1.08–10.52). Adults reporting a history of bloody urine or schistosomiasis were 4.36 times more likely to be sensitised to at least one allergen compared to those that did not (95% CI = 1.40–13.65). Artisanal miners were less likely to be atopic compared to other workers (OR = 0.56, 95% CI = 0.21–1.46). Based on the typical exposure profile of artisanal miners in Zimbabwe, this finding was unexpected. We conducted further analysis to compare artisanal miners with non-artisanal miners in the subgroup of adults with history of employment and the results are summarised in Table 6. The two groups shared similar characteristics with the exception that the majority of artisanal miners were male (70.27%) and more likely to consume alcohol (OR = 2.59, 95% CI = 1.07–6.25).

**Table 6:** Comparison of socio-demographic and health-related characteristics between artisanal miners and other occupations.

Characteristic	Artisanal miners (*n* = 37)	Other occupations (*n* = 116)	Total (*n* = 153)	OR(95% CI)
Socio-demographic characteristics				
Age(years), mean(sd)	43.24 (16.36)	46.43 (18)	45.66 (17.61)	1 (0.97–1.01)
**Sex, *n* (%)**				
Male	26 (70.27)	30 (25.86)	56 (36.60)*	Ref
Female	11 (29.73)	86 (74.14)	97 (63.40)	0.15 (0.07–0.33)
**Level of education, *n* (%)**				
No education	1 (2.70)	9 (7.76)	10 (6.54)	Ref
Primary	14 (37.84)	42 (36.21)	56 (36.60)	3 (0.35–25.82)
Secondary and tertiary	22 (59.46)	65 (56.03)	87 (56.86)	3.05 (0.36–25.42)
Ever smoked^a^	10 (27.30)	17 (14.66)	27 (17.65)	2.16 (0.89–5.25)
Passive smoking	20 (54.05)	49 (42.24)	69 (45.10)	1.61 (0.76–3.39)
Alcohol use	11 (29.73)	16 (14.04)	27 (17.88)*	2.59 (1.07–6.25)
Health related characteristics, *n* (%)				
Atopy	10 (30.30)	41 (42.27)	51 (39.23)	0.59 (0.26–1.38)
Polysensitisation				0.86 (0.67–1.11)
History of allergy among parents, n (%)	10 (27.03)	30 (25.86)	40 (26.14)	1.06 (0.46–2.45)
History of allergy among siblings, n (%)	9 (24.32)	43 (37.39)	52 (34.21)	0.54 (0.23–1.25)
Nasal allergy, n (%)	107 (28.01)	23 (21.3)	130 (26.53)	0.95 (0.43–2.09)
Skin allergy, n (%)	59 (15.49)	11 (10.19)	70 (14.31)	0.78 (0.29–2.10)
History of Tuberculosis	5 (13.51)	13 (11.21)	18 (11.76)	1.24 (0.41–3.74)
History of bloody urine/Schistosomiasis	6 (16.22)	16 (13.79)	22 (14.38)	1.21 (0.44–3.36)

*p < 0.05.

a *p = 0.086.*

## 4 Discussion

This study sought to identify the most important risk factors for atopy from a variety of self-reported environmental and lifestyle exposures among children and adults residing in a rural community in Zimbabwe. The overall prevalence of atopic sensitisation was 31.17% with a higher prevalence among adults (33.33%) compared to children (23.53%; *p* = 0.059). Self-reported nasal and skin allergies were more prevalent in adults than in children. The prevalence of allergic sensitisation in this study population was within the range (21.5–42.8%) of the prevalence of positive SPTs reported in studies from other African countries (Ghana, Uganda, Cameroon, and South Africa) that included children and adults from urban areas, rural areas or a combination of both communities (40–45).

### 4.1 Demographic and lifestyle characteristics

While there were no differences in age between atopic and non-atopic children, girls were significantly more likely to be atopic than boys (*p* = 0.04). Studies have reported that after puberty, girls seem to be at higher risk of developing atopic diseases than boys due to hormonal influences or sex-specific factors (57). Among adults, atopic individuals tended to be older than non-atopic individuals (*p* = 0.005), contrary to observations from studies in developed countries where atopy decreased with age (58). The likelihood of atopic sensitisation increased with increasing age among adults (OR = 1.02, 95%CI = 1.01–1.03). In addition to being older than the non-atopic adults, atopic adults were predominantly middle aged, with mean ages of 48.71 years (±20.03 years) and 46.10 years (±17.04 years) among men and women, respectively. There is relatively limited representation of middle-aged and advanced-aged individuals in the current body of work related to atopy and allergic diseases. Available data are mainly from studies in children, adolescents and young adults. As a steady increase in allergies has been noted in older adults and older individuals, future research should focus on this population group (59, 60). We also noted generational differences in demographic characteristics between the adults and children. For instance, adults were raised in larger families than were children, as reported by the significantly greater median number of siblings reported by adults (6, IQR = 4–8) than by children (4, IQR = 3–6; *p* < 0.05). There is substantial epidemiological evidence for the sibling effect, indicating the inverse relationship between the number of siblings and the risk of allergic sensitisation and disease among adults and children in various developing countries (61). However, there was no evidence of such a relationship in this study population.

There were 77 (20.16%) adults who reported consuming a predominantly traditional diet when asked to recall and describe their childhood dietary habits. However, a follow-up question to describe the type of food consumed over the last 12 months revealed that dietary habits have changed to reflect a more modern or processed diet. None of the children and only 9 (2.36%) adults said they currently consume a predominantly traditional diet. Another Zimbabwean study similarly reported low levels of daily traditional food consumption (9.3%) among adults, and the reasons cited included weak value chains, difficult and time-consuming cooking procedures for some food types, misconceptions about quality and safety, and aggressive commercial advertising of modern foods (49). Modern diets, characterised by the intake of highly processed foods with high energy, high saturated fat, high protein, low fibre and low levels of vitamins and minerals, have been implicated in increasing susceptibility to allergic diseases (62). Subsequently, the protective effects of nutrients such as antioxidants, oligosaccharides, polyunsaturated fatty acids, folate and other vitamins are an area of increasing research interest as a strategy for the prevention of allergic diseases (63, 64). Alcohol also appeared to increase the likelihood of atopy among adults in this study (OR = 1.88, 95% CI = 0.93–3.78), a finding that has been reported in other studies in Europe and Asia (65, 66). Although variables related to diet were not significant enough for inclusion in the current multivariate analysis, the ongoing nutrition transition in Sub Saharan Africa will likely continue to influence the development of allergic diseases and other non-communicable diseases (NCDs) (67). In Zimbabwe, there is renewed interest in traditional foods due to their perceived health benefits (49). Leveraging this attention to traditional foods in Zimbabwe to promote their consumption and to address the current barriers to access may be an important intervention for allergic disease prevention and control. A study in rural Crete found that a high level of adherence to the Mediterranean diet had a beneficial effect on allergic rhinitis, asthma-like symptoms and atopy. They concluded that a diet high in antioxidants may prevent the expression of allergic diseases in this population (68). It is plausible that a typical traditional Southern African diet could offer similar levels of protection, as there is evidence, though sparse, indicating high levels of bioactive compounds in indigenous fruits (69) and leafy vegetables (70).

### 4.2 Air quality

The relationship between air quality and allergy has received considerable attention. Therefore, various sources of both indoor and outdoor air pollutants, known to be important risk factors for allergic diseases, were considered in this study (71). Reports of dampness and mould inside the home were common, and these variables were included in the final models for both adults and children. Although not statistically significant, dampness and mould, as measured by self-reported history of water damage or roof leakage and the presence of visible mould on walls, appeared to increase the risk of allergic sensitisation among both children and adults. Housing conditions in low-income communities, if not properly maintained, may encourage the proliferation of microorganisms due to dampness and inadequate ventilation (72). In addition, 83% of participants reported that they practice indoor cooking using firewood, further compromising the indoor air quality (73). In Zimbabwe, women and children in rural communities have been reported to be particularly vulnerable to air pollutants from biomass combustion (74).

With an overall prevalence of passive smoking of 47.54%, exposure to environmental tobacco smoke (ETS) was quite common in this study population. Among children, passive smoking almost doubled the odds of atopic sensitisation (OR = 1.93, 95% CI = 0.70–5.30). In adults, parental smoking increased the odds of atopic sensitisation (OR = 1.54, 95% CI = 0.95–2.50). Exposure to environmental tobacco smoke (ETS), particularly among children and adolescents, has been associated with asthma and allergic rhinitis and influences sensitisation to both food and aeroallergens (75–78). Our results support the existing body of evidence against tobacco smoke, further emphasising the need for tobacco control interventions in the community to minimise exposure.

### 4.3 Infectious diseases and atopy

We explored the relationship between infectious diseases and atopy due to reports of schistosomiasisendemicity and high TB burden in Gwanda district (38, 45). A history of TB was reported by 34 (8.90%) adults and was also associated with atopic sensitisation among adults who had ever been employed (OR = 3.37, 95%CI = 1.08–10.52). There is evidence from observational studies of an inverse relationship between *Mycobacterium tuberculosis* infection and the presence of atopy or symptoms of allergic asthma (47, 79). This observation, however, is derived mostly from studies conducted in developed countries and among individuals with latent TB infection. On the other hand, our study participants had a previous history of active TB, which is immunologically different from latent infection (80). A study carried out in Peru to test whether a history of active TB was associated with atopy and allergic diseases found no evidence of a protective effect and concluded that the possibility of atopic disease in patients with a history of active tuberculosis could not be ignored (81).

Reports of history of bloody urine or schistosomiasis were also significantly more common in adults 32 (8.38%) than in children 2(1.85%). Adults reporting a history of bloody urine or schistosomiasis were twice as likely to be sensitised to at least one allergen compared to those who did not (*p* = 0.056). Furthermore, a history of bloody urine or schistosomiasis diagnosis was an even more influential risk factor for atopy among adults who had previously been employed (OR = 4.36, 95% CI = 1.40–13.65). Although a history of helminth infection was positively associated with atopy in our study, there is conflicting epidemiological evidence for this relationship, which has been attributed to the genetic susceptibility of the host and type of parasite as well as the timing, duration and intensity of infection (82–86). A recent meta-analysis, which included 80 studies, revealed that *Ascaris lumbricoides* infections were associated with an increased risk of atopy among helminth-infected adults (87). According to a study of newly arrived Ethiopian immigrants to Israel, a greater incidence of SPT positivity was observed after 1 year of follow-up in individuals who either remained infected (with lower intensity) or became infection free after treatment than in individuals with no evidence of infection (*p* = 0.045). Their conclusion that a changing burden of helminth infection coupled with exposure to new environmental factors might increase the likelihood of allergic sensitisation is a reasonable explanation for our findings (46). Another explanation we considered was the possibility of cross-reactivity between allergen extracts from mopane worms, mites and cockroaches and helminth antigens due to the presence of proteins such as tropomyosin and myosin (88). These findings further highlight the potential challenges in allergy diagnosis that may need to be addressed in areas endemic for infectious diseases.

### 4.4 Occupational exposures

Adults with work experience reported a wide range of occupations, such as artisanal mining, domestic work, farming, cross-border trading and construction. A number of these occupations have been documented to offer very little protection from harmful exposures and to contribute to the growing burden of environmental pollution in African communities (56). This includes uncontrolled or unregulated exposure to many irritant pollutants, such as dust, smoke and pesticides (33, 89). The risk and intensity of exposure to these harmful factors among different vulnerable population groups continue to increase due to the lack of clear regulatory policies, particularly in informal occupational settings (90, 91). The most frequently reported occupation in this study was artisanal mining 37 (24.34%). This was expected since mining is a major economic activity in Gwanda district because of the substantial mineral reserves (39). An increase in artisanal and small-scale miners in the area has had a significant negative effect on the environment and health of residents in the mining areas (39, 40). Having considered the potential vulnerability of this group, we carried out additional analysis to identify possible predictors of atopic sensitisation in the subpopulation of adults who reported having an occupation outside of the home in their lifetime. A history of TB and helminth infection were the most influential factors for atopy in this group. Infection with helminths and *Mycobacterium tuberculosis* in occupational settings is common in Zimbabwe and other sub-Saharan African countries (48, 92, 93). While infections have been found to have a protective effect according to the hygiene hypothesis, it seems that the environmental factors described in the Anthropocene might be more influential in promoting the development of atopy and allergic diseases in the presence of these infectious agents. In rural African communities, a steady increase in asthma and atopy has been observed despite the presence of protective factors associated with a rural lifestyle (94).

## 5 Strengths and limitations

A particular methodological strength of this study was the use of skin prick testing for the assessment of atopic sensitisation, for which the response rate was fairly high (93.15%). Additionally, a wide range of environmental exposures found in both childhood and adulthood were investigated using a collection of carefully selected questions that were a reflection of the study area’s environmental characteristics. A number of the factors reported, such as passive smoking, dampness and the presence of mould, are potentially modifiable; hence, these findings may be considered in the design and implementation of early public health interventions to prevent and control allergic diseases in the community. Nonetheless, our study is limited by its reliance on self-reports to collect environmental exposure information, which increases the vulnerability of the sample to recall bias. Every effort was made to ensure that most of the questions were extracted from validated questionnaires (43, 44, 95) and were sufficiently appropriate for children to minimise recall bias. In the questionnaire, we did not probe further about the duration of employment or the specific occupational exposures, thereby inherently introducing information bias. Additionally, the protective effects of artisanal mining and indoor cooking on atopy were unexpected and we believe that may have been due to the ‘healthy worker effect’, a form of selection bias (96). This underscores the need for longitudinal studies to explore these relationships. The use of non-probability sampling in the recruitment of participants could weaken the study as it potentially limits the generalizability of the study findings. This sampling approach was inevitable, as previously described in the feasibility study (18). Considering the ubiquitous nature of the environmental factors of interest in this study, we believe that the recruited participants provided a representative account of the community characteristics.

## 6 Conclusion

The prevalence of allergic sensitisation was comparable to that in other African countries. The findings of this study demonstrated that Gwanda district is characterised by several environmental factors that are known to influence the development of asthma and other allergic diseases. The risk factors included age, sex, passive smoking, alcohol consumption, history of bloody urine, TB, dampness and visible mould. The combination of previous TB or helminth infection and exposure to harmful pollutants typically found in occupational settings created the perfect storm of risk factors for atopy in adults. While most of these risk factors are similar to those found in developed countries, there are important exceptions, such as a history of active TB and previous helminth infection that justify future investigations. The presence of many modifiable risk factors further underscores the need to prioritise the early implementation of public health interventions to prevent and control the burden of allergic diseases. Designing longitudinal studies to explore the temporal and causal relationships between these factors and allergic outcomes is essential.

## Data Availability

The raw data supporting the conclusions of this article will be made available by the authors, without undue reservation.
